# Re: Infection control in burn patients: are fungal infections underestimated?

**DOI:** 10.1186/1757-7241-17-56

**Published:** 2009-10-31

**Authors:** David J Dries

**Affiliations:** 1 Regions Hospital, St Paul, MN, USA

## Abstract

A response to Struck MF. Infection control in burn patients: are fungal infections underestimated? Scand J Trauma Resusc Emerg Med. 2009 Oct 9;17(1):51. [Epub ahead of print] PubMed PMID: 19818134.

Dr. Struck [1] appropriately points out the importance of infecting agents apart from bacteria in the burn-injured patient. Burn patients are frequently cited as having the highest risk for invasive fungal infection as the burn wound provides an ideal portal for invasive infection while inducing immune dysfunction. Management of large burns exposes patients to risks identified in other patient groups including central venous lines, urinary catheters, prolonged mechanical ventilation and broad-spectrum antibiotics.

Unfortunately, it is difficult to determine the true incidence and significance of fungal infections in the burn population. Contamination of urine, respiratory tract and skin by organisms such as *Candida albicans *is extremely common. Criteria for identifying true infection in the setting of burns remain unclear. Clinical findings, such as fever, may not be discriminatory to help identify invasive infection in burn patients. Specific definitions for burn/wound infection rely heavily on wound appearance; fungal infection, in contrast, is notoriously difficult to diagnose on clinical findings alone. At present, a wide variety of practices exist among major North American burn centers to address this problem.

The *American Burn Association *recently published a review of burn patients with positive fungal cultures [2]. In all, positive cultures were seen in approximately 6% of 7,000 total admissions reviewed by reporting facilities. The incidence of positive fungal cultures varied widely, ranging from between 0.7% and 24% of patients treated at individual burn centers. There was no consistent pattern of treatment even if organisms were identified in the bloodstream. The majority of positive cultures came from the wound and respiratory tract (Figure 1).

**Figure 1:**
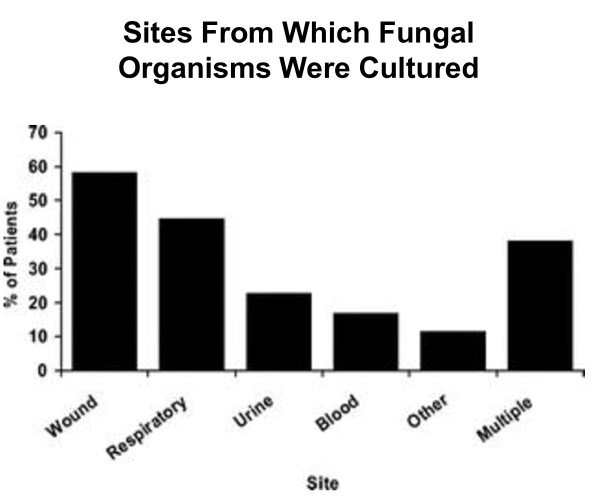
**Sites From Which Fungal Organisms Were Cultured**. © J Burn Care Res 2008; 29:213-221.

When logistic regression was employed to examine factors relating to mortality, age, burn size and inhalation injury showed positive correlation. A positive culture of mold or Aspergillus was also predictive of death. Each treated fungal culture was associated with an increased hospital length of stay by nearly eight days. Surprising in this data was a high use of TPN, immunosuppressive agents and the presence of malignancy. In summary, positive fungal cultures are common in burns. Clinical significance must be better defined. At present, there is no consistent indication for prophylaxis. Aggressive wound debridement and avoidance of central venous catheters, parenteral nutrition and other immunosuppressive agents as possible can be recommended.

In my practice, I will treat positive fungal blood cultures. I will not treat positive sputum cultures unless a quantitative threshold for pneumonia is reached. Finally, I do not consider prophylaxis given the equivocal impact on mortality unless a patient has multiple risk factors [3-5].
